# A Novel CPAP Device With an Integrated Oxygen Concentrator for Low Resource Countries: In Vitro Validation and Usability Test in Field

**DOI:** 10.1109/OJEMB.2024.3413574

**Published:** 2024-06-12

**Authors:** Poletto Sofia, Zannin Emanuela, Ghilotti Emanuele, Putoto Giovanni, Ichto Jerry, Lochoro Peter, Obizu Moses, Okori Samuel, Corno Matteo, Dellacà Raffaele

**Affiliations:** Electronics, Information and Bioengineering Department (DEIB)Politecnico di Milano 18981 20133 Milan Italy; Fondazione IRCCS San Gerardo dei Tintori 9265 20900 Monza Italy; Cuamm Medical Doctors for Africa 18317 35121 Padova Italy; St. John's XXIII Hospital of Atapara Aber 70134 Uganda

**Keywords:** Continuous positive airway pressure, low-and middle-income countries, respiratory distress syndrome, under-5 mortality, usability test

## Abstract

*Goal:* To develop and validatea novel neonatal non-invasive respiratory support device prototype designed to operate in low-resource settings. *Methods:* The device integrates a blower-based ventilator and a portable oxygen concentrator. A novel control algorithm was designed to achieve the desired fraction of inspired oxygen (FiO_2_) while minimizing power consumption. The accuracy of the delivered FiO_2_ and the device power consumption were evaluated in vitro, and a formative usability test was conducted in a rural hospital in Uganda. *Results:* The agreement between the set and delivered FiO2 was high (limit of agreement:−5.6 ÷ 3.8%). For FiO2 below 60%, the control algorithm reduced the power drain by 50%. The device was also appreciated by intended users. *Conclusion:* The prototype proved effective in delivering oxygen-enriched continuous positive airway pressure in the absence of compressed air and oxygen, holding promise for a sustainable and effective implementation of neonatal respiratory support in low-resource settings.

## I. Introduction

Every year, approximately 5.2 million children under 5 die, and 98% of these deaths occur in low- and middle-income countries (LMICs) [1], [2]. Approximately 46% of these deaths are related to hypoxemia or acute respiratory distress and could be prevented by a more spread and effective implementation of respiratory support [1], [3], [4].

Non-invasive respiratory support is relatively low-cost and feasible across a wide range of low-resource settings [6], [7]. In particular, nasal Continuous Positive Airway Pressure (nCPAP) is widely used to reduce the work of breathing and improve blood oxygenation [8].

There are several methods to generate CPAP [9], bubble CPAP (bCPAP) being the simplest and most common CPAP method in LMICs [10]. However, several factors still limit the widespread implementation of CPAP in LMICs, especially in peripheral centers [5]. Effective CPAP requires compressed medical air and oxygen. Standard CPAP systems use large bias flows and waste large amounts of compressed medical air and oxygen, which are expensive and limited in low-resource settings.

The most common sources of medical oxygen in LMICs are cylinders and oxygen concentrators. Cylinders are expensive, poorly sustainable and require a reliable distribution network, which is uncommon in LMICs [11]. Oxygen concentrators are cost-effective compared to cylinders but are cumbersome, need stable power sources, and typically cannot be used with standard ventilators [12], [13]. Moreover, connecting an oxygen concentrator to a ventilator's gas supply is ineffective for two reasons: first, a significant amount of oxygen is wasted through the expiratory pathway; second, standard oxygen concentrators cannot adjust oxygen production for patients needing low fractions of inspired oxygen, leading to energy waste and quicker deterioration of molecular sieves like zeolite.

Compressed medical air is less common than oxygen in LMICs. Therefore, inexpensive bCPAP systems that connect modified oxygen prongs and water bottles to a pure oxygen source are often used [5]. Even though these solutions are effective [10], they carry important limitations. Indeed, pure oxygen carries risks of oxygen toxicity, including but not limited to retinopathy of prematurity, and necessitates high oxygen flows to work properly [14]. CPAP devices specifically developed for low-resource settings are also available, but they either require an external source of oxygen or continuous stable electricity [20].

To overcome the abovementioned technical limitations to the safe and effective implementation of CPAP in low-resource settings, we developed a novel non-invasive neonatal mechanical ventilator independent from compressed medical air and oxygen sources. The device integrates a blower-based ventilation module and a small oxygen concentrator. The latter delivers an oxygen-enriched gas mixture directly at the inlet of the patient interface, avoiding oxygen waste through the expiratory pathway. Moreover, the oxygen concentrator is modulated based on the required fraction of inspired oxygen to produce only the needed amount of oxygen and minimize power consumption.

This paper presents 1) the design of the device, 2) its in vitro validation in terms of range and accuracy of the delivered fraction of inspired oxygen and power drain and 3) a formative field usability test.

## II. Materials and Methods

*Study design and setting*

The device design and development and the in vitro validation study were performed at the TechRes Lab, Politecnico di Milano.

### A. Device Development

#### 1) System Architecture

**Fig. 1.**
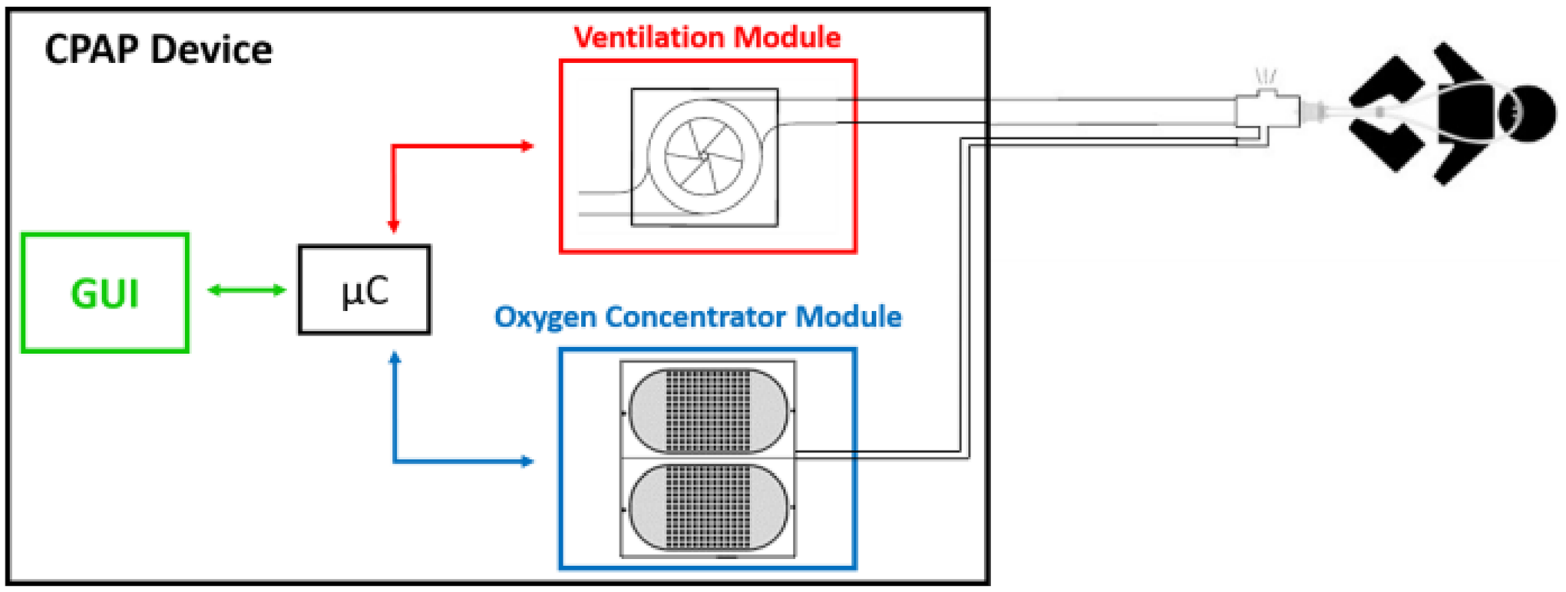
Schematic of the device. Red: ventilation module, blu; oxygen concentrator module; green: Graphic User Interface (GUI). The core microcontroller (µC) controls all the units.

The device is made of four modules: the ventilation platform, the oxygen concentrator, the Graphic User Interface (Supplementary Material), and the control unit (Fig. 1).

**Fig. 2.**
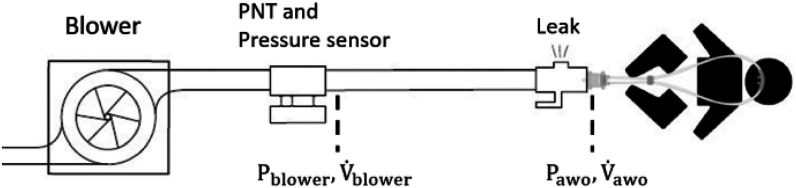
Structure of the ventilation module. PNT: pneumotachograph. P_blower_, ${\dot{V}} $_blower_: pressure and flow at the blower outlet. P_awo_, $\dot{V} $_awo_: pressure and flow at the patient's airways opening.

The ventilation module is based on a radial blower, while the oxygen concentrator produces an oxygen-enriched gas mixture that is blended with the air from the ventilation module to achieve the desired fraction of inspired oxygen at the patient interface. The oxygen-enriched gas mixture is injected between the intentional leak and the patient interface to minimize oxygen waste. An intuitive GUI allows the user to set the respiratory support settings, visualize the pressure and flow tracings, and manage the alarms. All the device units are controlled via a custom-made board, which hosts the main microcontroller (µC, STM32F746ZG; ST Microelectronics, Geneva, CH), the power drivers for actuators and the circuits for data communication with the sensors. This unit is powered by a switching 24 V AC/DC power supply (RSP-500-24; Mean Well, New Taipei, TW). A rechargeable lithium battery (24V DC-6000 mAh, Aftertech, Reggio Emilia, IT) guarantees a 30- to 60-minute backup.

#### 2) Ventilation Module

**Fig. 3.**
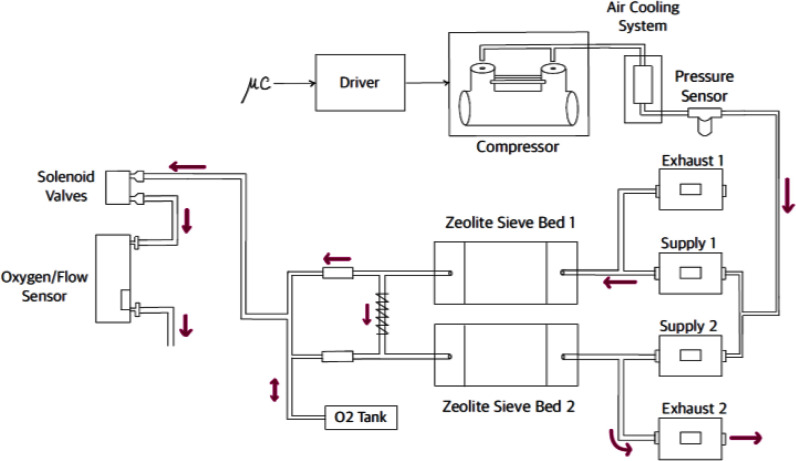
Schematic of the oxygen concentrator. The microcontroller drives the compressor, which pressurizes ambient air into the two zeolite sieve beds through two pairs of supply/exhaust valves. Two solenoid valves arranged in parallel control the output oxygen-enriched gas flow. An oxygen and flow sensor measures the oxygen concentration and gas flow at the output of the module. Purple arrows show the gas flow direction in case bed 1 is under adsorption and bed 2 under desorption.

Fig. 3 shows the structure of the ventilation module. The core is a mono-stage radial blower powered by a brushless DC motor with three Hall-effect sensors that allow highly accurate control of the rotor position (U65MN; Micronel, Tagelswangen, CH). Pressure and flow are measured at the blower outlet (${{\mathrm{P}}_{\text{blower}}}$, ${{\dot{\mathrm{V}}}_{\text{blower}}}$) using a ±75 mbar differential piezoresistive pressure transducer (HCLA0075B; First Sensor, Berlin, DE) and a custom-made 3D printed pneumotachograph (resistance ∼0.3 cmH_2_O·s/L) coupled with a ±2.5 mbar differential pressure transducer (HCLA02X5B; First Sensor, Berlin, DE). The breathing circuit has a single-limb configuration with an intentional leak that allows carbon dioxide washout and generates the desired pressure into the circuit. A customized 3D-printed connector incorporates an intentional leak and an inlet for the oxygen-enriched gas from the oxygen concentrator (Supplementary Fig. 5).

The intentional leak has a non-linear characteristic that allows the generation of 5 cmH_2_O of pressure with a bias flow of 3 L/min.

Pressure and flow sensors measure the variables at the inlet of the breathing circuit. The airway opening pressure and flow (${{\mathrm{P}}_{\text{awo}}}$and$\ {{\dot{\mathrm{V}}}_{\text{awo}}}$) are estimated based on the resistance of the single-limb breathing tube (R_tube_) and the flow through the intentional leak.

\begin{align*}
{{\mathrm{P}}_{\text{awo}}} =& {\mathrm{ }}{{\mathrm{P}}_{\text{blower}}}--{\mathrm{ }}{{\mathrm{R}}_{\text{tube}}}\,\,\,{{{\mathrm{\dot{V}}}}_{\text{blower}}} \tag{1}\\
{{{\mathrm{\dot{V}}}}_{\text{awo}}} =& {{{\mathrm{\dot{V}}}}_{\text{blower}}} - {{{\mathrm{\dot{V}}}}_{\text{leak}}} \tag{2}
\end{align*}

We approximated R_tube_ as a constant and the pressure-flow characteristic of the intentional leak as follows:

\begin{equation*}
{{{\mathrm{\dot{V}}}}_{\text{leak}}} = 0.013 \cdot \mathrm{P}_{\text{blower}}^{0.704} \tag{3}
\end{equation*}

Note that $\dot{\mathrm{V}} $_awo_ does not account for the flow coming from the oxygen concentrator.

We implemented a PID controller in the main microcontroller that uses the difference between the desired P_awo_ from the GUI and the measured P_awo_ as input to adjust the speed of the radial blower. The PID output is converted into an analog signal using a 12-bit Digital to Analog peripheral of the microcontroller and sent as target speed to a dedicated servo-controller (Escon module 50/5; Maxon Motor, Sachseln, CH) that implements a four-quadrant closed-loop control system of the blower speed.

#### 3) Oxygen Concentrator Module

Fig. 3 shows the schematic of the oxygen concentrator.

The oxygen concentrator operates on the Pressure Swing Adsorption (PSA) principle using two molecular sieves made of zeolites, porous materials that selectively adsorb nitrogen from ambient air when pressure increases [15]. In each cycle, one molecular sieve is in the adsorption stage (pressurized to produce an oxygen-enriched gas flow), while the other is in the desorption stage (depressurized to ambient pressure to release the previously adsorbed nitrogen). Part of the oxygen-enriched gas produced by the sieve in the adsorption phase is used to wash out the nitrogen from the sieve in the desorption phase to increase output oxygen concentration. In the subsequent cycle, the role of the two sieves is reversed.

**Fig. 4.**
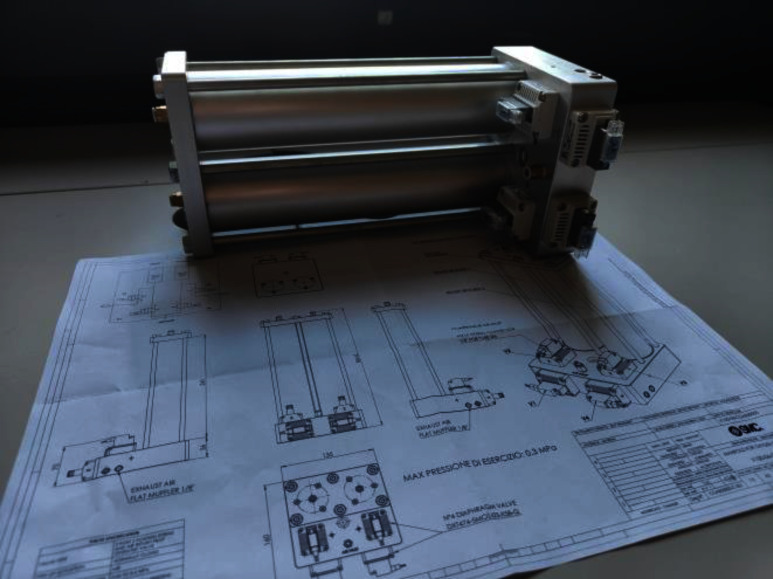
Oxygen concentrator manifold designed and produced by SMC Italy, Milano, Italy.

The oxygen concentrator core component is the compressor (BD-08AB-D; Shenzhen Boden Technology Development Co., Shenzhen, Guangdong, CH), an oil-free, brushless dual head medical air pump driven by its proprietary servo controller electronics with a speed set point set by a digital Pulse Width Modulation (PWM) signal. A copper tube and a small aluminium cylinder filled with alumina (100 ml) cool and dry the pressurized air. The air is driven alternatively in the two zeolite-filled (JLOX 101A; Luoyang Jalon Micro-nano New Materials Co., Yanshi, Henan, CN) 300 ml-molecular sieves. Each sieve is coupled with two diaphragm valves (DXT474; SMC, Tokyo, JP), called supply and exhaust valves. When a sieve is under adsorption, its supply valve is open, and its exhaust valve is closed. Conversely, when a sieve is in the desorption phase, its supply valve is closed, and its exhaust valve is open. The supply and exhaust valves switch every 3 seconds. A custom manifold, engineered by SMC Italy, integrates the sieves, valves, and all connections, enhancing robustness and minimizing potential connection errors during manufacturing and maintenance (Fig. 4).

The oxygen-enriched gas produced by the sieves is stored in a 170 ml reservoir acting as low-pass filter to provide a continuous gas source and delivered to the patient through two Solenoid Valves (SV) arranged in parallel (K8; Camozzi Automation, Brescia, IT). The valves opening is controlled by adjusting the duty cycle of a 50-Hz PWM driving signal, which presents a hysteresis-free linear relationship with the output flow.

The oxygen flow the concentrator needs to produce to achieve the requested fraction of inspired oxygen (FiO_2 TARGET_) at the airway opening is computed using mass balance equation:

\begin{equation*}
\text{Fi}{{\mathrm{O}}_{2{\mathrm{\ TARGET}}}} = \frac{{{{\mathrm{O}}_{2{\mathrm{\ CONC}}}} \cdot {{{{\mathrm{\dot{V}}}}}_{\text{CONC}}} + 0.21 \cdot {{{{\mathrm{\dot{V}}}}}_{\text{awo}}}}}{{{{{{\mathrm{\dot{V}}}}}_{{\mathrm{CONC\ }}}} + \ {{{{\mathrm{\dot{V}}}}}_{\text{awo}}}}} \tag{4}
\end{equation*}

Where ${\dot{\mathrm{{V}}}_{\text{awo}}}$ is the ventilator flow that reaches the airways opening (3), $ \dot{\mathrm{V}}_{{\mathrm{CONC\ }}}$ the concentrator flow, and ${{\mathrm{O}}_{2{\mathrm{\ CONC}}}}$ the oxygen concentration. Multiple combinations of ${{\dot{\mathrm{V}}}}_{{\mathrm{CONC\ }}}$ and ${{\mathrm{O}}_{2{\mathrm{\ CONC}}}}$will yield the same FiO_2 TARGET_. This defines an under-constrained problem or, in other words, a control allocation problem. The controller exploits this degree of freedom to minimize power consumption by solving the following constrained static minimization problem:

\begin{align*}
& {\mathop{\min }_{\text{CS},\text{SV}}} {{\mathrm{P}}_{{\mathrm{CONC\ }}}}\left( {\text{CS},\text{SV}} \right) \tag{5}\\
& \text{s.t.} \frac{{{{\mathrm{O}}_{2{\mathrm{\ CONC}}}} \cdot {{{{\mathrm{\dot{V}}}}}_{\text{CONC}}} + 0.21 \cdot {{{{\mathrm{\dot{V}}}}}_{\text{awo}}}}}{{{{{{\mathrm{\dot{V}}}}}_{{\mathrm{CONC\ }}}} + \ {{{{\mathrm{\dot{V}}}}}_{\text{awo}}}}} = \ \text{Fi}{{\mathrm{O}}_{2{\mathrm{\ TARGET}}}} \tag{6}
\end{align*}

The solution to the above problem requires the knowledge of three characteristic relationships:

${{\mathrm{P}}_{{\mathrm{CONC\ }}}}( {\text{CS},\text{SV}}), {\dot{\mathrm{V}}}_{\text{CONC}} ({\text{CS},\text{SV}}) \,{\mathrm{and}}\, {{\mathrm{O}}_{2{\mathrm{ CONC}}}} ( {\text{CS},\text{SV}} )$ i.e., the dependence of the concentrator flow, oxygen concentration and compressor power on the two control variables: the compressor speed (CS) and solenoid valves (SV) duty cycles.

A principle model of these relationships would depend on many physical parameters that are difficult to measure or estimate. Therefore, we used a data-driven modelling approach: a static model was derived by sampling the input space and measuring the resulting oxygen concentration $({{\mathrm{O}}_{2{\mathrm{\ CONC}}}}),$ flow (${\dot{\mathrm{V}}}_{\text{CONC}}$)and absorbed power. In a series of experiments, the CS was sampled from 20% to 100% in steps of 10% and the SV from 10% to 100% in steps of 10.

Multiple combinations of CS and SV yield the same (${{\mathrm{O}}_{2{\mathrm{\ CONC}}}},$) flow (${{\dot{\mathrm{V}}}}_{\text{CONC}} $) confirming the bi-dimensional dependency (see Section III). The CS and SV characteristics were fitted with the following polynomial expressions:

\begin{align*}
 \text{CS} =& {{\mathrm{p}}_{00}} + \ {{\mathrm{p}}_{10}} \cdot {{{\mathrm{\dot{V}}}}_{\text{CONC}}} + {{\mathrm{p}}_{01}} \cdot {{\mathrm{O}}_{2{\mathrm{\ CONC}}}} + {{\mathrm{p}}_{20}} \cdot {{{\mathrm{\dot{V}}}}_{\text{CONC}}}^2 \\
& + {{\mathrm{p}}_{11}} \cdot {{{\mathrm{\dot{V}}}}_{\text{CONC}}} \cdot {{\mathrm{O}}_{2{\mathrm{\ CONC}}}} + {{\mathrm{p}}_{02}} \cdot {{\mathrm{O}}_{2{\mathrm{\ CONC}}}}^2 \tag{7}\\
 \text{SV} =& {{\mathrm{p}}_{00}} + \ {{\mathrm{p}}_{10}} \cdot \text{CS} + {{\mathrm{p}}_{01}} \cdot \ {{{\mathrm{\dot{V}}}}_{\text{CONC}}} + {{\mathrm{p}}_{20}} \cdot \mathrm{C}{{\mathrm{S}}^2} \\
& + {{\mathrm{p}}_{11}} \cdot {{{\mathrm{\dot{V}}}}_{\text{CONC}}} \cdot \text{CS} + {{\mathrm{p}}_{02}} \cdot {{{\mathrm{\dot{V}}}}_{\text{CONC}}}^2 \tag{8}
\end{align*}

Note that for ease of use in the following computation, (7) and (8) describe the inverted ${{\mathrm{P}}_{{\mathrm{CONC\ }}}}({\text{CS},\text{SV}}),\ {{\dot{\mathrm{V}}}}_{\text{CONC}} ( {\text{CS},\text{SV}} )$ characteristics. The parameters of the polynomial fitting are shown in Supplementary Table I, the simple one-dimensional monotonic relationship between CS and power consumption in Supplementary Fig. 6.

Based on the three identified functions, the minimization problem (5) is solved with the following algorithm:

1) Compute ${{\dot{\mathrm{V}}}}_{\text{awo}}$=${{\dot{\mathrm{V}}}}_{\text{blower}}$–${{\dot{\mathrm{V}}}}_{\text{leak}}$,
2) Solve and sample (4) to find *N* (${{\mathrm{O}}_{2{\mathrm{\ CONC}}}},\ {{\dot{\mathrm{V}}}}_{\text{CONC}})$ candidates.
3) For each (${{\mathrm{O}}_{2{\mathrm{\ CONC}}}},\ {{\dot{\mathrm{V}}}}_{\text{CONC}})$ solve (7) and (8) to get (CS, SV) within the CS and SV actuation limits.
4) Choose, among the feasible (CS, SV) pairs, the power-minimizing one, i.e., the one with the lowest CS.

The solution to such a static optimization problem is precomputed and stored in a look-up table for a more efficient implementation.

The oxygen-enriched gas injection from the concentrator acts as a disturbance in the bandwidth of the P_awo_ controller. The blower pressure sensor measures this effect, and the PID controller rejects the disturbance.

### B. Validation Protocol

The in vitro validation aimed to evaluate the accuracy of the FiO_2_ delivered at the airways opening while quantifying the device power drain.

#### 1) Experimental Set-Up

**Fig. 5.**
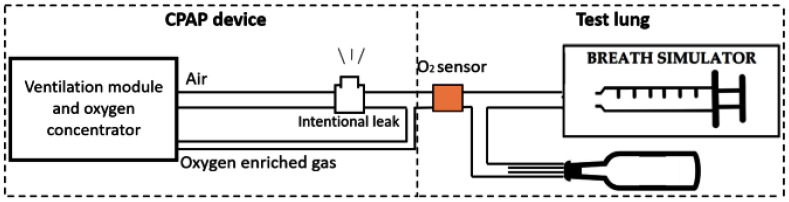
In vitro validation experimental set-up. The CPAP device is attached to an active test lung with known mechanical properties (R = 60 cmH_2_O*s/L, C = 0.0015 L/cmH_2_O). A paramagnetic oxygen sensor is placed at the mouthpiece to measure the oxygen reaching the test lung.

Fig. 5 shows a schematic of the validation set-up:

An active test lung was realized using a mechanical analogue of the respiratory system of either preterm or respiratory distressed infants (R = 60 cmH_2_O*s/L, C = 0.0015 L/cmH_2_O) [19] arranged in parallel with a breath simulator driven by volume tracings recorded on human infants to reproduce realistic respiratory patterns. A paramagnetic oxygen sensor (Paracube Sprint Sensor; Hummingbird Sensing Technology, Crowborough, U.K.) measured the FiO_2_ at the patient interface. The speed of the oxygen concentrator compressor was recorded for the whole duration of the test and used to compute the overall power drain.

#### 2) Experimental Protocol

The accuracy of the delivered FiO_2_ and the corresponding power consumption were evaluated for all combinations of the following settings:

• CPAP at 3, 5, 8 and 10 cmH_2_O;
• FiO_2_ from 25% to 70% in steps of 5%;
• simulated minute ventilation of 800, 1000, and 1500 ml/min.

#### 3) Data Analysis

We used Bland-Altman analysis to compare the target and measured FiO_2_ at the inlet of the breath simulator. We evaluated the power drain at different FiO_2 TARGET_, comparing it with the maximum power consumption of the oxygen concentrator.

### C. Feasibility Study

A field usability study was performed at St. John's XXIII Hospital of Atapara, Aber, Uganda (Supplementary Material). Following a 20-min training, 15 nurses were asked to perform six tasks on a mannequin and to fill the Post-Study System Usability Questionnaire (PSSUQ). For each task, we recorded the number and type of misuses.

## III. Results

*Oxygen concentrator characterization*

The power drain of the concentrator components was:

– supply and exhaust valves: < 1W each;
– solenoid valves delivering oxygen to the patient: 1.9 to 5 W depending on their opening level;
– sensors: 10 W in total;
– compressor: 65 to 146 W, proportional to its speed see Supplementary Material.

**Fig. 6.**
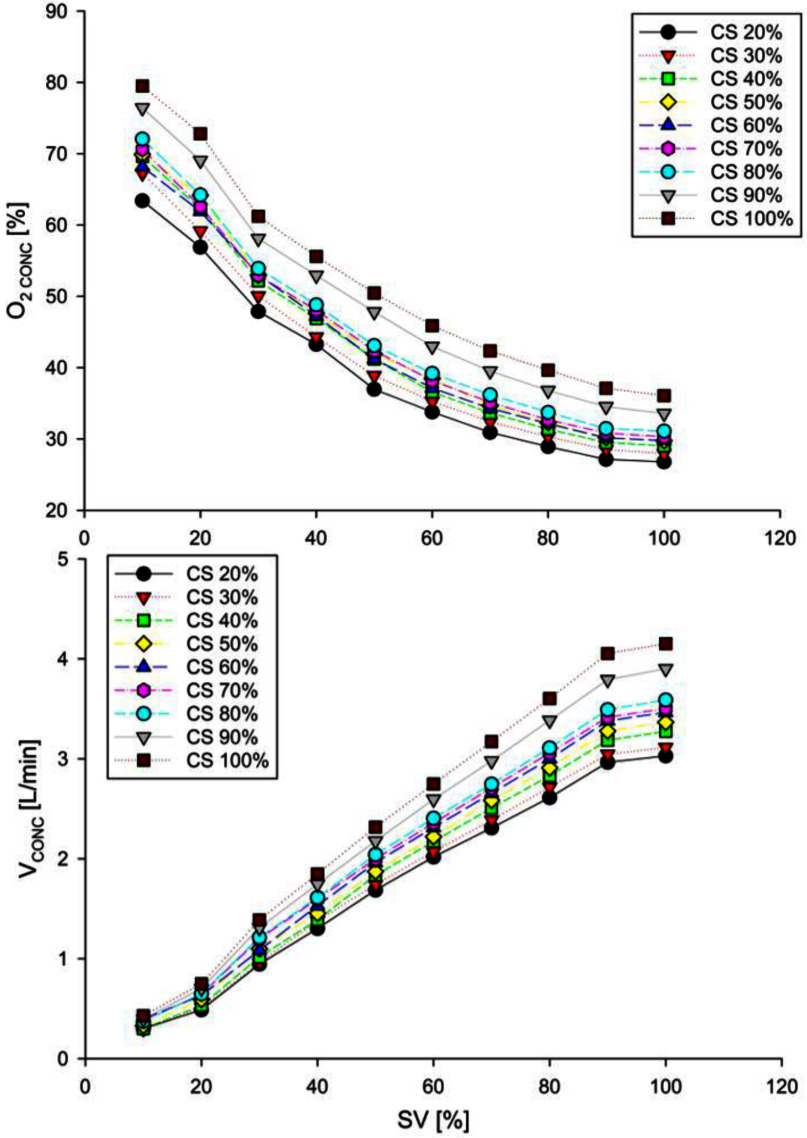
Results of the oxygen concentrator characterization. [top] Increasing solenoid valves' duty cycle decreases the oxygen concentration of the output gas flow; [bottom] Increasing solenoid valves' duty cycle improves the output gas flow. A higher compressor speed increases both the oxygen concentration (top) and flow (bottom) of the output.

Fig. 6 shows the flow and oxygen concentration of the oxygen-enriched gas for each combination of compressor speed and output valves aperture.

The flow and oxygen concentration of the output gas increased as the compressor speed increased. By contrast, as the valves aperture increased, the output flow increased, but oxygen concentration decreased.

We used such results to derive (7) and (8) coefficients.

*In vitro validation*

**Fig.7.**
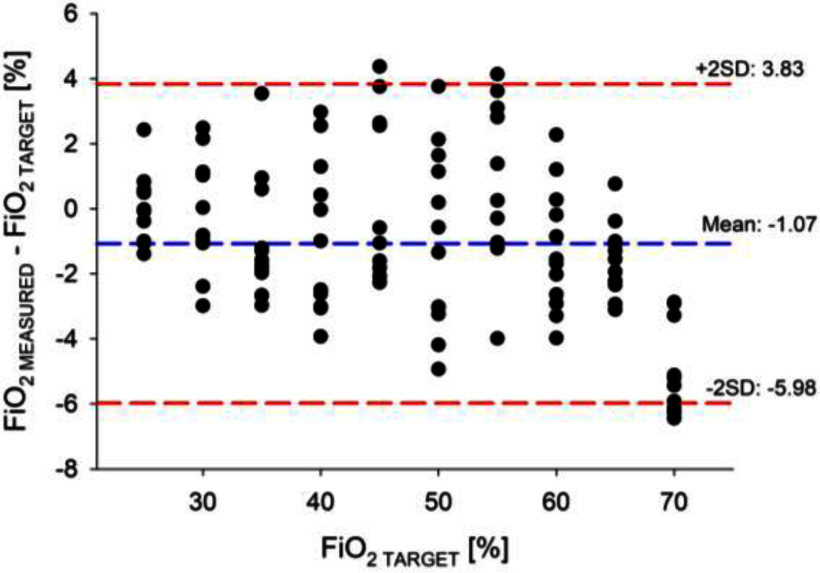
Bland-Altman plot showing the difference between delivered and set FiO_2_.

We found a strong agreement between the set and delivered FiO_2_ (Fig. 7): the mean difference was −1.07 %, and the limits of agreement were −5.56 and 3.83%.

**Fig. 8.**
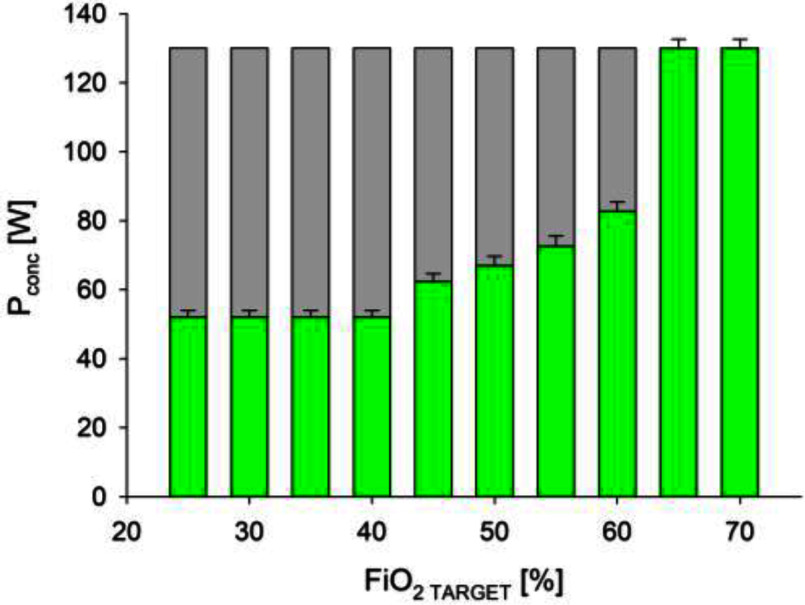
Power consumption of the compressor at different FiO_2_ levels. The automatic algorithm yields a power saving up to 50% when patients are requiring FiO_2_ equal or lower than 60%.

The oxygen concentrator power drain was 130 W in standard mode, producing the maximum achievable oxygen concentration. Thanks to the novel control algorithm, the power consumption decreased with FiO_2_, allowing a saving of up to 50% when the set FiO_2_ was below 60% (Fig. 8). Moreover, in these optimized conditions the back-up battery duration is more than 60 minutes.

*Feasibility study*

Participants made a total of 5 mistakes, 3 of which in the start-up phase. The mean±SD PSSUQ score was 1.64 ± 0.49 (worst 7), showing high appreciation.

## IV. Discussion

We described a novel device designed to provide non-invasive respiratory support to neonates in low-resource settings. The device integrates a turbine-operated CPAP module and a portable oxygen concentrator, resulting in independence from external sources of compressed air and oxygen, which are limited in LMICs [9]. The turbine ensures the possibility of providing CPAP at the desired FiO_2_, which is crucial to mitigate the risks of oxygen toxicity. For example, the unrestricted use of oxygen in preterm newborns can lead to the development of retinopathy of prematurity, which has a higher incidence in LMICs than in high-income countries[17], [18]. Compressed medical air is even more limited than oxygen in low-resource settings, and air-oxygen blenders are often too expensive [9]. Oxygen concentrators represent the most cost-effective and sustainable oxygen source in LMICs. However, oxygen concentrators usually fail to produce the high oxygen pressures and flow rates required by most standard blenders and respiratory support devices. Since neonatal respiratory support devices work with high bias flows, blending oxygen with air at the inlet of the breathing circuit results in a large waste of oxygen, making the treatment expensive and inefficient. The proposed system delivers an oxygen-enriched gas mixture directly at the airways opening, avoiding oxygen waste through the expiratory pathway. A novel characteristic of this device is that the oxygen concentrator speed is adjusted to achieve the desired FiO_2_, minimizing power consumption. Indeed, a major limitation of bedside oxygen concentrators is that they require an uninterrupted energy source and have high power consumption, which can be a bottleneck for their use in settings with unstable electricity. By contrast, thanks to the synergistic operation of the ventilation and oxygen concentrator modules, the proposed device has limited power drain and could potentially work on batteries.

Both the compressor speed and the opening of the output valves influence the oxygen concentration and the output flow. To achieve the desired FiO^2^, our algorithm automatically selects the optimal combination of oxygen concentrations and gas flows, minimizing power consumption. Since compressor speed primarily influences power usage, the oxygen concentrator control system determines the minimum speed required to attain the desired FiO2 and respiratory support settings. Compared to standard operation, the proposed approach decreases power consumption by 40 to 60%.

Devices specifically developed for LMICs are are already used to deliver bCPAP to neonates in resource-limited settings. Specifically, the Pumani (Hadleigh Health Technologies, US) and the Dolphin (MTTS, Vietnam) have an integrated blower but rely on an external oxygen source. The Vayu bCPAP (Vayu Global Health Innovations, US) employ an inexpensive gas entrainment blender but requires a source of compressed oxygen. Finally, the Baby CPAP (Diamedica, U.K.) have integrated sources of oxygen and air but has similar power consumption as bedside oxygen concentrators and, consequently, requires reliable electricity [9], [20].

The in vitro validation showed that the device can generate FiO_2_ levels up to 70% with minimal errors at all considered CPAP levels. The inability to achieve FiO_2_ close to 100% was deemed acceptable, considering its intended use for treating neonates with mild to moderate respiratory distress in LMICs. We speculate that patients with severe respiratory failure requiring FiO_2_ above 70% cannot be cared for in centres with limited facilities and resources because they need high-intensity treatments (e.g., invasive mechanical ventilation) and close monitoring delivered by skilled personnel. Even if the device can function with minimal facilities, its safe and effective use necessitates healthcare professionals' ability to identify patients who may benefit, monitor them, adjust respiratory support settings accordingly, and manage the airway interface. Patients should receive good essential newborn care for the device to be cost-effective. Otherwise, non-respiratory issues might negatively affect the outcome.

The present results are encouraging and justify further development and technology transfer of the device, incorporating the inputs and suggestions of the usability study, thanks to the partnership with a social enterprise with strong expertise in developing technologies for low-resource countries. Clinical studies on neonates are warranted to evaluate the safety and effectiveness of the device, along with its reliability on prolonged use in clinical practice in a relevant environment.

## V. Conclusion

We developed a proof-of-concept respiratory support device for treating neonates with mild to moderate respiratory distress in low-resource settings. The device does not need external medical gasses and has limited power consumption. It could accurately deliver CPAP at FiO_2_ up to 70% on a test lung mimicking infants up to 10 kg. If such results are confirmed in clinical field studies, after further development, the proposed device can positively impact the respiratory outcomes of infants with respiratory distress in low-resource settings.

## Supplementary Materials

See Supplementary materials to find further images which give more details on the proposed device as well as a detailed description of the Guided User Interface and of the performed usability study on field.

## Conflict of Interest

The authors declare that they have no competing interests.

Authors contributions

**Conceived the study**: SP, EZ, MC, RD.

**Critically revised the device specifications**: GP, JI, PL, MO, SO,

**Design of the device**: SP, EG, EZ, RD.

**In-vitro validation study**: SP, EG.

**Usability study protocol and data collection**: SP, GP, JI, PL, MO, SO, RD.

**Data analysis**: SP, EZ, EG, MC, RD.

SP, EZ, MC, RD drafted the manuscript. All authors reviewed and revised the manuscript and have read and approved its final version.

## Supplementary Materials

Supplementary materials
