# Supplementary material for: A Novel CPAP Device With an Integrated Oxygen Concentrator for Low Resource Countries: In Vitro Validation and Usability Test in Field
Source: IEEE Open J Eng Med Biol. 2024 Jun 12;5:498–504. doi: 10.1109/OJEMB.2024.3413574 (PMC11268943; doi:10.1109/OJEMB.2024.3413574)
Supplement: Supplementary materials [file supp1-3413574.pdf]

Supplementary Materials

A Novel CPAP Device with an Integrated Oxygen Concentrator for Low Resource Countries: In Vitro Validation and Usability Test in Field

Poletto Sofia, *M.Sc*; Zannin Emanuela, *PhD*; Ghilotti Emanuele, *M.Sc*; Putoto Giovanni, *MD*; Ichto Jerry, *MD*; Lochoro Peter, *MD*; Obizu Moses, *Dott*; Okori Samuel, *MD*; Corno Matteo, *Prof*; Dellacà Raffaele, *Prof*

In the supplementary material of this paper, we present 1) the design of the novel mechanical ventilation device Graphical User Interface 2) the results of the device field usability test.

Graphical User Interface

We developed a Graphical User Interface (GUI) that allows real-time data visualization, setting of ventilation parameters, and alarm management (Supplementary Figure 1). The GUI was implemented on a Raspberry Pi board (Raspberry Pi 4; Cambridge, UK) with an integrated 7’’LCD touchscreen using Electron, a free and open-source software framework hosted and developed by GitHub. A rotary knob was also introduced as an alternative to the touch screen to allow more user-friendly screen navigation in some conditions, for example, when the operator’s hands are wet.

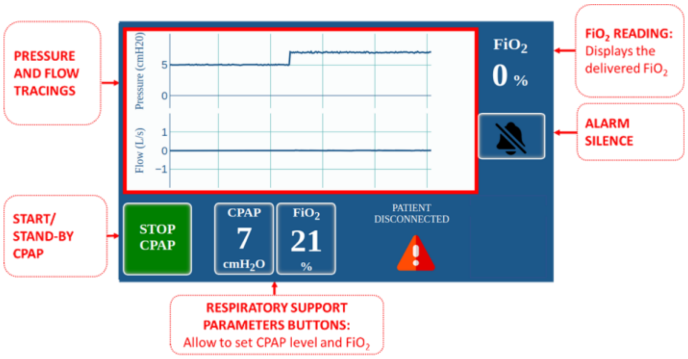

Supplementary Fig. 1. GUI. The graphs display the real-time tracings of the pressure and flow at the airways opening; the button in the bottom-left corner of the screen allows to start and stop CPAP; the CPAP and FiO_2 buttons at the bottom of the screen allow to set the desired CPAP and FiO_2 levels; the FiO_2 indicator at the upper-right corner of the screen shows the FiO_2 that is delivered, and the bell symbol allows to silence the alarms.

When the user touches one of the two buttons at the bottom of the screen, a pop-up window allows the user to modify the settings and confirm the action before changing the system operation (Supplementary Figure 2).

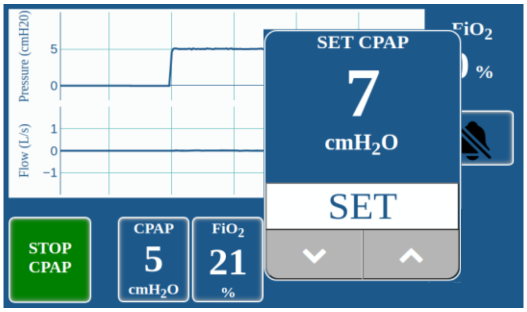

Supplementary Fig. 2. Example of a GUI pop-up that allows changing the pressure support settings.

An audible tone and a frame (red or yellow, depending on the alarm priority) surrounding the graphs indicate one of the following alarm conditions: patient disconnection, high or low ventilation pressure, tube occlusion, and low FiO_2. A written message is also displayed, describing the alarm condition. By touching the bell symbol on the right-hand side of the screen, the audible tone is silenced for 30 seconds.

Feasibility study

We enrolled healthcare personnel working in the neonatal intensive care unit at St. John XXIII Hospital of Atapara (Aber, Uganda) and having experience in using respiratory support devices. The most common therapy used by the interviewed personnel to treat respiratory distress was oxygen delivery through nasal prongs connected to oxygen concentrators. In the hospital there were also two Dolphin CPAP (MTTS-Asia, Hanoi, VN), which were scarcely used because of the need of an external source of compressed oxygen, which was not available most of the time. They received the device user manual and 20-min training consisting of three videos presenting:

- a short introduction to the use CPAP in infants;
- the main characteristics of the novel CPAP device;
- a tutorial on how to use the device.

After the training session, participants had 30 minutes to familiarize themselves with the device without supervision. A printed quick reference guide (see online supplementary material) was attached to the back of the device, along with a list of the possible alarms and relative solutions.

The usability test consisted of six tasks:

a. Start-up: the user had to set-up the breathing circuit and turn the device on.

b. Respiratory support settings recognition: the user had to fill a chart with the CPAP and FiO_2 set on the ventilator.
c. Parameters changing: the user had to adjust the CPAP and FiO_2 to pre-specified values using the touchscreen and the rotary knob.
d. Alarms identification: the user had to recognize different alarms and react to them.
e. Breathing circuit checking: the user had to check the breathing circuit and patient interface and identify possible errors.
f. Shut down: the user had to turn off the device and disconnect all the breathing circuit components.

Each user performed the six tasks on their own. For each task, the number and type of misuses were recorded. At the end of the test, each participant filled out a post-study system usability questionnaire (PSSUQ), a 16-question self-reported subjective evaluation of perceived system usability (Supplementary Figure 3). PSSUQ scores ranged from 1 to 7, with lower scores representing better perceived usability [16].

Post-Study System Usability Questionnaire
Q1. Overall, I am satisfied with how easy it is to use this system.
Q2. It was simple to use this system.
Q3. I was able to complete the tasks and scenarios quickly using this system.
Q4. I felt comfortable using this system.
Q5. It was easy to learn to use this system.
Q6. I believe I could become productive quickly using this system.
Q7. The system gave errar messages that clearly told me how to fix problems.
Q8. Whenever I made a mistake using the system, I could recover easily and quickly.
Q9. The information material provided with this system was clear.
Q10. It was easy to find the information I needed.
Q11. The information was effective in helping me complete the tasks and scenarios.
Q12. The organization of information on the system screens was clear.
Q13. The interface of this system was pleasant.
Q14. I Liked using the interface of this system.
Q15. This system has all the functions and capabilities I expect it to have.
Q16. Overall, I am satisfied with this system.

Supplementary Fig. 3. Post-Study System Usability Questionnaire.

Fifteen nurses working in St. John's XXIII Hospital of Atapara (Aber, Uganda) participated in the field usability study. Participants made a total of 5 errors, three during the device start-up, one in the alarms' identification and one in the ventilatory circuit leakage check. In particular, the main challenges were found in assembling the breathing circuit due to the presence of temperature probes and heater wires whose electrical plugs were not clearly identified on the device. The mean PSSUQ score was 1.64 ± 0.49, showing high appreciation for the device (Supplementary Figure 4).

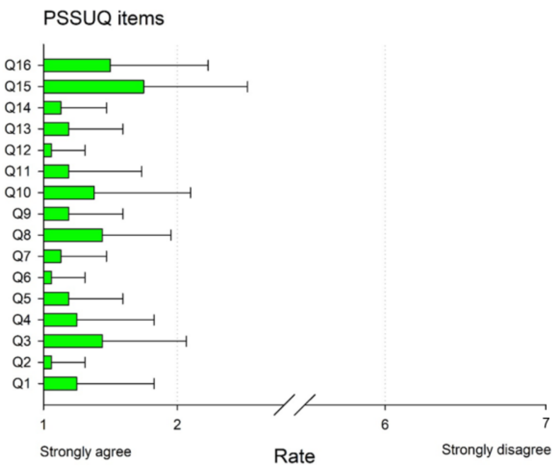

Supplementary Fig. 1. PSSUQ results (1=high appreciation, 7=low appreciation). All participants showed high appreciation for the device, giving a mean mark for each question lower than 2.

The field usability study performed in a rural hospital in Uganda showed that, after a short training, the users were able to complete the most relevant tasks, including setting up the device, modifying the settings, and recognizing and managing the alarms. The users reported a high appreciation of the device, as assessed with a validated usability scale. The collected free feedback provided during the interviews with the subjects identified possible improvements for making our device more useful and effective in these settings. In particular, all subjects asked to have a pulse oximeter integrated in the device, to be able to monitor saturation in real-time and adjust CPAP parameters more effectively. Another important feedback concerned the breathing circuit design. We were asked to make it easier to mount, having temperature probes and heater wire already plugged to the device, just requiring the nurse to connect them to the breathing tube.

Limitations of the study include the limited number of subjects and the single-centre design of the usability study, which might limit the generalizability of the results. Moreover, the usability study was conducted using a mannequin and not on patients, limiting the possibility of evaluating the effectiveness and efficacy of the device to achieve specific goals in the intended environment. The present usability study was a formative field study aimed at collecting needs, suggestions and feedback from potential users in a co-design approach to the design and development of an industrial device. Further summative studies, including more tasks and diverse users, are necessary for regulatory purposes. In the present study, the validation was performed in vitro, while the performance of the device in the clinical setting is likely affected by its interaction with the patient. However, using the device in a clinical study at the current stage would not have been ethical. Indeed, it is important to notice that the proposed device does not have regulatory approvals yet, which are crucial for the safe and effective application of medical devices in clinical practice and, as such, to contribute to the progress in neonatal care.

In conclusion, the preliminary field usability study showed that the device is easy to learn and use, and potential users highly appreciated it.

Supplementary Images

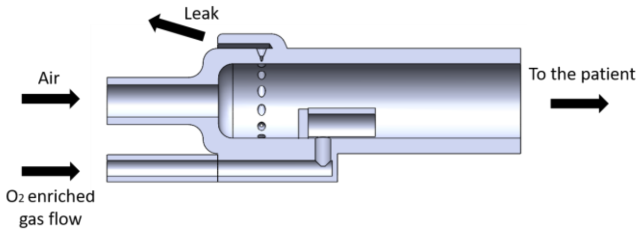

Supplementary Fig. 5. Section of the oxygen connector integrating the intentional leak (holes around the connector protected by a circular ring for avoiding possible accidental obstruction), the oxygen-enriched gas and air inlets, and the outlet to the patient.

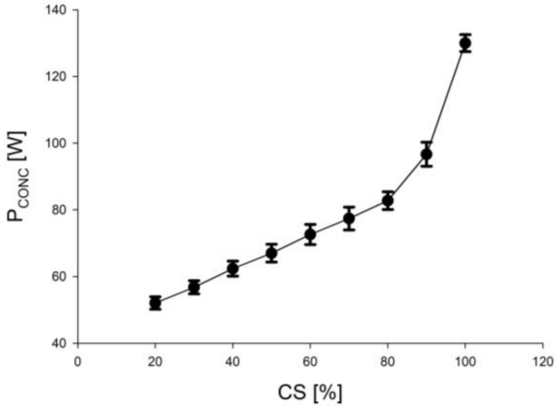

Supplementary Fig. 6. Relationship between the duty cycle of the compressor PWM driving signal (e.g., a proxy of CS) and power consumption.

	Equation 7	Equation 8
**p_{00}**	-500.40	2.07
**p_{10}**	154.40	0.10
**p_{01}**	11.57	26.98
**p_{20}**	-15.00	0
**p_{11}**	-0.87	-0.12
**p_{02}**	-0.06	1.80

Supplementary Table 1. Estimated values of the coefficients of equation (7) and (8). $R^2 = 0.90$ for (7) and $R^2 = 0.99$ for (8).
